# Happy Hosts? Hedonic and Eudaimonic Wellbeing in the Sharing Economy

**DOI:** 10.3389/fpsyg.2022.802101

**Published:** 2022-03-16

**Authors:** Georg von Richthofen

**Affiliations:** Alexander von Humboldt Institute for Internet and Society (HIIG), Berlin, Germany

**Keywords:** sharing economy, wellbeing, peer-to-peer accommodation, Airbnb, hosts, hospitality, customer misbehavior

## Abstract

Sharing economy platforms mediate exchanges between service providers and consumers. The experiences of service providers in the sharing economy have been extensively studied. Nevertheless, our knowledge in regard to the extent to which providers’ participation influences their wellbeing remains incomplete. This study focuses on the peer-to-peer accommodation platform Airbnb and explores why and how different aspects involved in hosting can contribute to or hinder hosts’ hedonic and eudaimonic wellbeing. To that end, I conducted a netnography and depth interviews with Airbnb hosts. Based on a qualitative analysis of the overall dataset, I identify three sources of positive affect associated with hosting, namely, the sociability involved in the host-guest interaction, the act of providing hospitality, and positive feedback from guests. However, I also identify four conditions, which can turn hosting into a source of negative affect, namely, customer misbehavior, high volumes of guests, negative reviews, and income dependency. In addition, I elaborate on the relationship between hosting and life satisfaction in regard to the income that hosts generate through hosting and the working conditions of Airbnb hosts. Last but not least, I show that being a provider on Airbnb can contribute to (and in some cases hinder) eudaimonic wellbeing, focusing on four dimensions of eudaimonia, namely, self-realization, personal growth, a sense of purpose and meaning, and relationships. Theoretical and managerial implications for service providers and sharing economy platforms are discussed.

## Introduction

The sharing economy has been defined as a “scalable socioeconomic system that employs technology-enabled platforms to provide users with temporary access to tangible and intangible resources that may be crowdsourced” (Eckhardt et al., 2019, p. 3). According to this definition, the sharing economy includes firms such as Bird and Zipcar that provide consumers temporary access to their own resources (Bardhi et al., 2012). In addition, the sharing economy includes firms such as Airbnb, BlaBlaCar, and Uber that use digital platforms to match consumers, who need some resource (e.g., an accommodation or a car) or service (e.g., hospitality or a ride) with external providers. This study focuses on the latter category that Eckhardt et al. (2019) classify as archetypical sharing economy businesses.

Archetypical sharing economy businesses rely on external service providers as their co-producers (Dellaert, 2019). To attract service providers, these businesses often articulate a compelling mission (Benoit et al., 2017) and highlight benefits of participating in their ecosystems. Airbnb, for example, showcases happy hosts welcoming their guests with a smile in its advertisements and stresses that hosting can be a lucrative, pleasant, and transformative experience that contributes positively to hosts’ lives. Similarly, ride-hailing platforms such as Lyft emphasizes the fun involved in driving and getting to know strangers during rides (Freiherr von Richthofen, 2019).

Popular brands such as Airbnb and Uber have been extremely successful in attracting providers to their platforms. According to the statistics published on the Airbnb website, for instance, more than 4 million hosts use the Airbnb platform to rent more than 5.6 million listings (Airbnb, n.d.). Albeit impressive, these numbers give us no insight into the actual experiences of service providers in the sharing economy. Is participating in the sharing economy really as appealing and beneficial as the advertisements of platforms collectively seem to suggest? Moreover, how does participating in the sharing economy impact the wellbeing of service providers? Taking stock of the sharing economy literature, Eckhardt et al. (2019, p. 15) emphasize that “the relationship between sharing economy participation and happiness is an intriguing issue,” which requires further exploration. The goal of this article is to help address this gap in the literature, by focusing on the peer-to-peer accommodation platform Airbnb.

Founded in 2010 in the aftermath of the financial crisis, Airbnb became virtually synonymous with the sharing economy (Schor, 2016). While researchers have studied the platform extensively (Dann et al., 2019), only a fraction of this literature focuses on the actors who provide accommodations on the platform, commonly referred to as “hosts.” Initially, researchers were mainly focused on hosts’ participation motives as well as their experiences in the sharing economy. More recently, researchers began to analyze both the positive and negative consequences of hosting (Zhang et al., 2019; Buhalis et al., 2020). With the exception of one recently published study (Buhalis et al., 2020), none of these studies focused explicitly on the extent to which participating in the sharing economy contributes to or hinders hosts’ wellbeing. However, an analysis of the Airbnb literature indicates a number of outcomes which can be related to various dimensions of wellbeing. For example, studies show that the host-guest interaction can be a source of enjoyment (Ikkala and Lampinen, 2015; Farmaki and Kaniadakis, 2020), a positive affect associated with hedonic wellbeing (Ryan and Deci, 2001). Moreover, studies indicate that some hosts experience personal growth (Zhang et al., 2019) and derive meaning from hosting (Fitzmaurice et al., 2020; Makkar and Yap, 2020), both components of eudaimonic wellbeing (Ryff and Singer, 2008). Yet, there is also evidence that hosting can negatively impact hosts’ wellbeing (Zhang et al., 2019; Buhalis et al., 2020). For example, studies showed that some hosts had experiences which likely had a negative impact on their wellbeing, including but not limited to guests intruding on their privacy, damaging their properties, or even engaging in forms of sexual harassment (Zhang et al., 2019; Buhalis et al., 2020). In addition, some hosts experience emotional stress over guest reviews (Zhang et al., 2019), especially those who depend on hosting for their livelihood (Buhalis et al., 2020).

In this study, I will build on this rich interdisciplinary literature and an in-depth inductive analysis of interviews and forum posts by Airbnb hosts to address the following research question: Why and how do different aspects involved in hosting contribute to or hinder hosts’ wellbeing? In regard to hedonic wellbeing, I identify several sources of positive affect involved in hosting, such as sociability, hospitality, and positive feedback, as well as conditions which can reverse these positive influences or lead to negative affect, namely, customer misbehavior, high volume of guests, negative reviews, and income dependency. In addition, I elaborate on factors involved in hosting, which may potentially impact hosts’ life satisfaction, such as income and the working conditions of hosts. Second, I show why hosting can be a source of eudaimonic wellbeing, focusing on four associated dimensions, namely, (1) self-realization, (2) personal growth, (3) purpose and meaning, and (4) social relations. However, I also elaborate on some of the experiences and developments that can hinder eudaimonic wellbeing. For example, the interaction with guests tend to contribute to hosts’ social wellbeing, but hosting can also destabilize existing social bonds, most notably with neighbors bothered by the intrusions caused by guests. Finally, I discuss theoretical and managerial implications for service providers and sharing economy platforms.

The remainder of this study is organized as follows. First, I elaborate on the enabling concepts of this article, namely, hedonic and eudaimonic wellbeing and provide some background on the motives, types, and experiences of Airbnb hosts. Then, I describe the data and methods used in this paper, present the findings, and discuss their theoretical and managerial implications.

## Theoretical Background

### Wellbeing

In the positive psychology literature, wellbeing refers to an individualized and subjectively experienced way of being, which depends both on behaviors and such objective circumstances as one’s health and social environment (De Vos et al., 2013; Prayag et al., 2021). In psychology, scholars commonly distinguish two related concepts of wellbeing—hedonic and eudaimonic wellbeing (Ryan and Deci, 2001). Hedonic wellbeing relates to the attainment of pleasure and avoidance of pain (Ryan and Deci, 2001); it is usually assessed by using three components: life satisfaction, the presence of positive affect, and the absence of negative affect (Diener et al., 1999).

Eudaimonic wellbeing, in contrast, focuses on the actualization of human potential, of fulfilling one’s true nature (Ryan and Deci, 2001). This involves identifying one’s potential strengths and limitations and choosing pursuits which provide personal meaning and purpose (Waterman et al., 2010). In other words, the goal of eudaimonic wellbeing is to pursue self-realization by “striving toward excellence based on one’s unique potential” (Ryff and Singer, 2008, p. 14). The implication of eudaimonic wellbeing implies “living a good life, not just a pleasant one” (Fisher, 2014). Psychologists have conceptualized eudaimonic wellbeing in a variety ways and developed several scales to measure its underlying dimensions. According to Ryff (1989), eudaimonic wellbeing consists of and can be assessed using six dimensions, namely, autonomy, personal growth, self-acceptance, life purpose, environmental mastery, and positive relationships. Waterman et al.’s (2010) alternative scale consists of the following six dimensions: self-discovery, perceived development of one’s best potentials, a sense of purpose and meaning in life, intense involvement in activities, investment of significant effort, and enjoyment of activities as personally expressive.

The relationship between hedonic and eudaimonic wellbeing continues to be debated (Diener et al., 2018). Ryan and Deci (2001, p. 148) argued that wellbeing is ultimately “probably best conceived as multidimensional phenomenon that includes aspects of both the hedonic and eudaimonic conceptions of wellbeing.” Therefore, I will explore the experiences of Airbnb hosts both from a hedonic and an eudaimonic perspective. To that end, I will draw on the concepts and dimensions that psychologists use to assess hedonic (Diener et al., 1999) and eudaimonic (Ryff, 1989; Waterman et al., 2010) wellbeing. However, I will not use the scales that psychologists developed for data collection. Instead, I will use the aforementioned concepts (e.g., eudaimonic wellbeing) and dimensions (e.g., personal growth) as enabling lens (e.g., Figueiredo et al., 2016) to guide the analysis and interpretation of the qualitative data collected for this study.

### Motives, Types, and Experiences of Airbnb Hosts

The motives of service providers in the sharing economy are diverse and encompasses the entire spectrum of utilitarian to altruistic motives (Bucher et al., 2016). After a multitude of studies on the motives of Airbnb hosts, we know that their main motive to engage on the platform is to make money. However, many Airbnb hosts are also motivated by the social benefits associated with hosting (Ikkala and Lampinen, 2015; Lampinen and Cheshire, 2016; Zhang et al., 2019; Farmaki et al., 2020). Social benefits involve the possibility to interact and socialize with guests, learn about different countries and cultures, and form friendships. A third motive of Airbnb hosts worth mentioning concerns the flexibility that platform work offers to hosts (Lampinen and Cheshire, 2016; Zhang et al., 2019; Farmaki et al., 2020).

Recent research indicates that this multiplicity of motives can be associated to some extent with different types of hosts (Farmaki et al., 2019; Farmaki and Kaniadakis, 2020). Farmaki et al. (2019) posit that Airbnb hosts can be categorized into professional and non-professional hosts. Professional hosts are those who tend to manage single or multiple properties. For them, hosting represents an important and sometimes the main source of income. Non-professional hosts tend to share their home (i.e., rent a room), and appear to be primarily motivated by the social benefits of hosting. Refining this typology further, Farmaki and Kaniadakis (2020) identify four types of hosts: emerging professional economically– driven hosts, with many property listings and/or managing others’ properties; individual economically–driven hosts, with one or two property listings; individual economically-driven hosts, sharing their property; individual socially-oriented hosts, sharing their property.

While the motives of hosts have been extensively studied, our knowledge about the actual experiences of Airbnb hosts and long-term effects of hosting is more limited. Recent studies focused more explicitly on the outcomes of hosting (e.g., Zhang et al., 2019; Buhalis et al., 2020). Buhalis et al. (2020) identify a number of benefits and costs associated with being an Airbnb hosts. While they identified positive aspects such as income, companionship, meeting people, and taking pride in hospitality, they also observed negative aspects such as pressure to achieve high scores, unrealistic expectations by guests, damages, sexual harassment, and problems with neighbors. Zhang et al. (2019) identify five positive outcomes of hosting (cultural learning, financial gains, social connections, personal growth, feeling of achievement) and a number of negative outcomes such as risks, lack of privacy, and emotional stress about guests’ reviews. Throughout the finding’s section, I will relate to these and other findings, while exploring the experiences of hosts from a wellbeing perspective.

## Data and Method

Given the explorative nature of this study, I opted for a qualitative research design. I used a combination of a netnography and depth interviews to gain an in-depth understanding of the experiences of Airbnb hosts from a wellbeing perspective. Next, I will describe each method and the data collected using these methods separately.

### Netnography

Netnography is a research approach that applies ethnography to online environments (Kozinets, 2002). It has been widely adopted across the social sciences (Kozinets, 2015) and also been used to assess the impacts of hosting on Airbnb hosts (Buhalis et al., 2020).

Kozinets (2015) outlined several criteria for choosing netnographic field sites. For instance, sites should be relevant to the research question, active, and data rich. For this study, I conducted a netnography of the Airhostsforum^1^. The Airhostsforum fulfills most the criteria outlined by Kozinets (2015). Created in 2014 by Airbnb hosts and “dedicated to connecting hosts with other hosts” (Airhostsforum, n.d.), it is an active online community, which hundreds of hosts use to socialize with each other, share experiences and best practices, and discuss relevant developments. According to the statistics provided on the forum, it has several hundred active users per month. Overall, more than 373 thousand posts have been made to more than 14 thousand topics around hosting (Airhostsforum, n.d.).

My netnographic participation involved immersing myself in the forum from May 2015 to May 2018 following the guidelines of Kozinets (2015). Netnographic participation can involve different levels of engagement from reading posts to offering comments to becoming an organizer within the community (Kozinets, 2015). Given that I do not use Airbnb as a host myself, I decided to take a less participative stance (c.f., Kozinets and Handelman, 2004). After posting once on the forum to inform its members about my research, I engaged in several activities outlined by Kozinets (2015), namely, reading current messages regularly and in real-time several times a week, reading archives of messages, and following links to other pages. In addition, I engaged in conversations *via* personal messages and Skype with two members of the community. I also interviewed one of the two forum members formally.

Throughout my fieldwork, I archived conversations and quotes with relevance to my research question, that is, conversations and posts that helped me understand why and how different aspects involved in hosting contribute to or hinder hosts’ wellbeing. More specifically, I archived posts in which hosts reported positive or negative experiences related to hosting, because such posts can be indicative of pleasure and pain and thus of hedonic wellbeing. In addition, I also archived conversations and posts in which hosts reflected on the impacts that hosting has had on them and their lives, because such posts can be indicative of changes in life satisfaction and eudaimonic wellbeing. The data set from this field work encompasses more than 5,000 single-spaced pages of text. The netnographic data gave me rich insight into the experiences of Airbnb hosts. In relationship to the host-guest interaction, for example, consistent with the prior literature (e.g., Ikkala and Lampinen, 2015), I initially found that hosts generally enjoy the sociability involved in hosting and performing the role of the host (see section “Hosting as a Source of Positive Affect”). Reading through forum conversations about the negative aspects of hosting, however, I noticed that the level of enjoyment depends on a number of conditions, such as the behavior and volume of guests (see section “Hosting as a Source of Negative Affect”).

### Interviews

In addition to the netnography, I conducted depth interviews with Airbnb hosts. I adopted a purposive sampling approach to select interview participants based on three pre-defined criteria (Etikan et al., 2016). First, I selected hosts who were active on the Airbnb platform at the time of the interview (i.e., had listed accommodations on the platform). Second, I only interviewed hosts who were willing to share their perceptions (Farmaki et al., 2020). Third, I selected hosts who had hosted a number of guests that allowed them to assess both the benefits and costs of being an Airbnb host. While the participant with the most experience had hosted several hundred guests, the informant with the least experience hosted 30 guests. In total, I collected 11 in-depth interviews with Airbnb hosts between January 2015 and November 2018. I recruited eight participants through my personal network. I did not know any of the participants prior to the interview. Moreover, I interviewed two hosts after staying with them in their accommodations. In addition, I interviewed one host from the Airhostsforum. I stopped interviewing additional hosts, when interviews no longer yielded new relevant information (Fusch and Ness, 2015). The interviews were conducted in informants’ homes (4), at university (4), and over Skype (3). More information on the interview participants is provided in Supplementary Table 1.

The procedure of the interviews followed established guidelines for conducting consumer interviews (Belk et al., 2013; Arsel, 2017). Accordingly, I began the interviews with grand tour questions (McCracken, 1988), such as “How come you starting hosting on Airbnb?” and “How do you interact with your guests?” These questions then served as a starting point for further probes (Arsel, 2017). Later in the interview, I asked participants more specific questions about positive and negative experiences they had and about their routines as hosts. Supplementary Table 2 provides more detail on the interview protocol and the questions asked. Interviews lasted between 55 and 107 min, were taped, transcribed verbatim, and resulted in 206 single-spaced pages of text.

### Data Analysis

My data analysis was guided by the procedures outlined by Belk et al. (2013). Thus, I initially read all material multiple times to familiarize myself with the overall data set. In a second step, I coded the data set in view to the research question using Microsoft Word, iterating back and forth between the data as well as the literatures on wellbeing and the sharing economy. In regard to hedonic wellbeing, for example, I initially openly coded quotes illustrative of pleasure and positive affect, pain and negative affect, and life satisfaction. This yielded first descriptive codes such “guests writing notes of appreciation.” In a next step, I grouped these codes in an iterative process into more abstract clusters (Miles and Huberman, 1994), such as “sociability” as well as “positive” and “negative feedback.” To illustrate the iterative coding process in more detail, consider how the theme “customer misbehavior” emerged from my analysis. During the initial open coding process, I identified various cases, in which hosts reported negative experiences due to the behavior of guests. This yielded first codes such as “guest smoking inside the accommodation,” “guest inviting additional guests to the accommodation,” “sexual harassment,” “guest ignoring spatial boundaries,” “vandalism,” and “guest treating host in an impolite manner.” Moreover, I learned from immersing myself in the Airbnb literature that other researchers had made similar observations (Zhang et al., 2019; Buhalis et al., 2020). Buhalis et al. (2020), for example, found that some hosts complained about constant disruptions and requests by guests. In several rounds of coding, I collapsed the aforementioned descriptive codes into broader codes such as “guest breaking house rules.” Eventually, I realized that all of the aforementioned cases can be broadly categorized as instances of “customer misbehavior” (Harris and Reynolds, 2004). In the next section, I will present a detailed account of the findings of my analysis.

## Findings

In the findings, I will examine why and how different aspects involved in hosting can contribute to or hinder hosts’ hedonic and eudaimonic wellbeing. I will consider each perspective in turn.

### Hosting From the Perspective of Hedonic Wellbeing

In this section, I will present initial evidence which indicates that hosting can lead to both the experience of positive as well as negative affect and impact life satisfaction.

#### Hosting as a Source of Positive Affect

Being an Airbnb host can be associated with the experience of positive and pleasant emotional feelings and moods. Three sources of positive affect were especially salient in my data: (1) the sociability involved in the host-guest interaction, (2) the act of providing hospitality, and (3) positive feedback from guests. I will consider each source in turn.

First and foremost, studies show that Airbnb hosts value the social interaction with guests (Ikkala and Lampinen, 2015; Zhang et al., 2019; Buhalis et al., 2020; Farmaki and Kaniadakis, 2020). Notably, Ikkala and Lampinen (2015) found that the sociality involved in hosting can be best understood within the framework of “sociability”—“a form of sociality that gains its value from the interaction itself.” Similarly, my analysis shows that socializing can be a source of pleasure and positive affect. Consider Sophie, who lives with her husband and her two children in a home they bought a few years ago in Toronto:

I love the meeting, you know, being like “Why are you here?” and this window into their life, (…) this quick kind of vision of someone who has a totally different life from you and it’s very. It’s freeing, you know, it takes me out of my day, out of my own worries, to have for a moment a talk to someone else (…) (Sophie).

The quote reveals that Sophie not only enjoys meeting her guests (“I love the meeting”), but that these relatively brief social encounters also energize and stimulate her. Such interactions also enable her to escape her everyday life: “It’s freeing, you know, it takes me … out of my own worries”.

Second, research shows that Airbnb hosts tend to derive “gratification from being good hosts” (Lampinen and Cheshire, 2016). Similarly, Buhalis et al. (2020) found that hosts take pride in providing hospitality and showing guests their local culture. Consistent with these studies, I found numerous cases in which hosts expressed delight about being able to take care of their guests and being good hosts. One interview participant explicitly articulated that seeing guests having a good time is part of the reason why he and his wife enjoy being Airbnb hosts:

We also enjoy it when guests have a good time. When they are like: “Hey, the lake was really cool.” When you simply notice that they had a great evening and have just a wonderful time staying with you. That’s also something beautiful, where you receive an immaterial reward. (…) Yeah, that’s (…) part of the gratification. (Christian)

The third source of positive emotions and moods relates to positive feedback from guests. Review systems are key governance mechanisms of platform markets in general (Tadelis, 2016) and sharing economy platforms such as Airbnb in particular (von Richthofen and von Wangenheim, 2021). Studies found that hosts enjoy receiving positive feedback from their guests, be it in the form of personal notes guests leave behind or in the form of reviews (Ikkala and Lampinen, 2015; Wilkinson and Wilkinson, 2018). My own fieldwork confirms their finding. Several interview participants reported they enjoy reading positive reviews by their guests. Consider the following interview quote for example:

Interviewer: How come you started doing Airbnb?

   Jennifer: (…) I think the main motive, the main motivation wasn’t money, it was getting to know the people and also, I think you get to a period in your Ph.D. when, you know, you don’t get a lot praise, (…) you just, you get praise once a year, (…) and having a very good customer feedback (…) is really motivating in life.

The quote indicates that positive feedback by guests may even contribute to hosts’ life satisfaction, which I will discuss in section “Hosting and Life Satisfaction” in more detail. Collectively, the findings presented so far suggest that certain aspects of the hosting experience (sociability, hospitality, and positive feedback) can trigger pleasure and positive affect.

#### Hosting as a Source of Negative Affect

Despite its potential to contribute positively to the mood of Airbnb hosts, under certain conditions, hosting can turn into a source of pain and negative affect. My data analysis indicates that four conditions seem especially relevant in this regard: (1) customer misbehavior, (2) high volumes of guests, (3) negative reviews, and (4) income dependency. I will consider each condition in turn.

First, hosting can cause hosts to experience negative affect when guests engage in customer misbehavior (Harris and Reynolds, 2004). These misbehaviors, which largely mirror those of hotel industry guests, entail a range of behaviors including making unreasonable requests, breaking house rules, infringing on privacy, damaging premises, and sexual harassment (Zhang et al., 2019; Buhalis et al., 2020). Relative to the misbehavior of guests in hotels, however, the significance of such misbehaviors is additionally amplified when they takes place in providers’ own homes, a space that is supposed to be safe haven from the outside world (Mallet, 2004). In my interviews, I learned about various forms of customer misbehavior. In one case, a guest ignored the house rules by inviting an additional guest without notice, and by smoking inside the guest room. In another, a host was harassed by one of her guests. Moreover, in a third instance, the guests not only used the host’s apartment to hold a party in her absence, but they also stole some of her possessions.

Second, my analysis indicates that hosts’ experience depends to some extent on the volume of guests they host and the turnover they have. While new hosts who have guests occasionally very much enjoy performing the role of the host and interacting with their guests (Ikkala and Lampinen, 2015), I found that hosts who have a lot of turnover enjoy these interactions less over time. It seems as if having a lot of guests makes it more difficult for hosts to sustain the “semblance of hospitality” when they interact with their guests (von Richthofen and Fischer, 2019), which involves displaying the appropriate personal front (Goffman, 1959), by being cheerful and smiling (Darke and Gurney, 2000). Under such circumstances, performing the role of the host means that providers display feelings they don’t experience as authentic in these moments, that is, they engage in emotional labor (Hochschild, 1979; Bucher et al., 2020). Consider the following forum post for example:

(…) When my guests arrive, (…) I open the door, smile warmly and say, “Hello! You must be Susan. Come on in! I’m Barbara,” as I extend my hand to shake theirs (…) Of course, much of this is nothing more than an act, especially at the end of the season. I’m super nice and friendly to guest’s faces, when in reality I want to put part of the soundtrack of The Amityville Horror on a loop and play it. “GET OUUUTTTTTT! (…) (Chloe, September 12, 2018, reply to “3 misconceptions about hosting I learned this summer”).

Chloe is no exception. Even hosts who genuinely enjoy interacting with guests struggle to perform their roles toward the end of a busy season or a longer stay.

Third, I found that just as positive feedback can be a source of happiness, especially critical or negative feedback can be a source of negative affect. I repeatedly found that hosts were distraught by negative reviews, especially in cases where they felt unjustly evaluated. Several forum members used the Airhostsforum to seek emotional support after a negative review. As one host comments: “I tell myself to not let them (negative reviews) get to me, but they always do to some degree” (dcross9999, May 2, 2017, reply to “How do you let go of the negative reviews?”).

Last but not least, consistent with Schor et al. (2020), I found that the extent to which hosts enjoy hosting depends to some degree on whether they depend on the platform income. The reason for this seems to be that income dependency puts additional pressure on hosts to ensure guests are satisfied with their stay. Consider how investing more in her Airbnb changed the way Sarah felt about hosting:

(…) And then in October we decided to invest the money (…). It’s became more serious; it became more like a business. (…) In a way, it became less fun, I became much more anxious about the noise, I am always telling my kids you know shh shh shh, because it gets really loud down there, the main guest room is under our kitchen so we really try to get the kitchen done early in the evening so not doing that while the guests are there. So I feel a lot more pressure, I feel a lot more stress about them (the guests) being happy. Um. So it was a little more fun in the beginning when (…) it was just like (…) a buck you know. (Sarah).

The quote indicates that when hosts depend on the Airbnb income, they strive even more to provide a good experience to guests, which in some cases means that they restrict themselves more in their own home (see also section “Working Conditions”). Similarly, studies show that hosts, who depend on the Airbnb income, are emotionally stressed by the pressure to score high reviews, given that positive reviews are critical to secure future bookings (Zhang et al., 2019; Buhalis et al., 2020). On the Airhostsforum there is an entire topic, in which several hosts discuss feeling anxious about reviews, as the following statement illustrates: “I also always feel a bit anxious even though we have always had good reviews” (Shanghai, December 2015).

In sum, it emerges from my findings that the extent to which participating on the Airbnb platform leads hosts to experience pleasure and positive affect or pain and negative affect depends on several conditions and factors, not all of which are under the control of Airbnb hosts. Next, I will focus on life satisfaction, another component of hedonic wellbeing.

#### Hosting and Life Satisfaction

Life satisfaction refers to people’s “explicit and conscious evaluation of their lives, often based on factors that the individual deems relevant” (Diener et al., 2018, p.3). To date, the literature provides insufficient insight into the relationship between the participation of Airbnb hosts in the sharing economy and their life satisfaction. My own data analysis was inconclusive. My netnographic work, for example, yielded few posts which may be interpreted as explicitly related to life satisfaction. The following forum post is a rare exception in terms of its explicitness:

I can say that Airbnb changed my life for the better. Of course it’s a job, but in addition it is very rewarding emotionally and monetary. This week and it’s not even over yet, I made 500$. Where else I could make this much money working literally 3 h changing sheets and doing little cleaning and on top of that practicing my Spanish with non-English speakers from Venezuela FOR FREE!! I had couple of fiascos in a beginning, and they are very frustrating, to the point that I wanted to quit hosting. (…) (Yana, November 5, 2015).

The post seems to point to some factors that tend to be correlated with life satisfaction, such as social relationships as well as income (Diener et al., 2018). Moreover, it also points the working conditions of being an Airbnb host, a factor that is more specific to the peoples’ satisfaction in the workplace (Fisher, 2014). In the next two sections, I will discuss two aspects associated with hosting that seem to have the capacity to impact life satisfaction. To avoid being redundant, I will not iterate the role of the social benefits involved in hosting. Instead, I will focus on the role of the financial income generated through Airbnb and the working conditions of Airbnb hosts.

##### Income

Studies show that making money is the main reason why people list accommodations on Airbnb (Dann et al., 2019; Guttentag, 2019) and that the income generated through hosting can provide the foundation for a more comfortable lifestyle (Lampinen and Cheshire, 2016; Schor et al., 2020). Lampinen and Cheshire (2016), for example, found that hosts use the money they make for a variety of purposes: as supplemental income to help pay their rent, to finance their education, to cover unexpected medical expenses, or to have extra spending money. In finding that hosts use their earnings “to pay off educational debt, finance luxury spending (such as a spectacular wedding), or travel”, Schor et al. (2020, p.845) posit that platforms such as Airbnb add to providers’ “economic security and sense of agency and enable lifestyles that they could not otherwise afford.” Similarly, my interview participants use the money they make as Airbnb hosts to afford a larger apartment, to avoid taking in permanent tenants or roommates, to pay back their mortgages, to finance their own travels, or to splurge on hobbies.

However, over the years a more complex image has started to emerge. Mirroring the financial opportunity, hosting also constitutes a financial risk. Buhalis et al. (2020, p. 696) note that hosts find it increasingly difficult to meet the rising expectations of guests and that some hosts “fear for their livelihood and often for mortgages that they took to build their properties” and that this dynamic “exerts pressure to invest more in their service and over-perform without a fair return” (see also Farmaki and Kaniadakis, 2020). Similarly, I found that hosts occasionally question the profitability of their engagement on the Airbnb platform. The financial risk associated with Airbnb became evident to all hosts after the outbreak of the Corona pandemic, with many hosts struggling to make ends meet and cover costs associated with their rentals (Farmaki et al., 2020).

##### Working Conditions

Schor et al. (2020) found that hosting constitutes a highly appealing form of labor relative to other forms of gig work such as ride-hailing, because it required relatively little effort by comparison and can result in significant earnings, since consumers pay primarily for access to hosts’ properties. However, these insights are arguably biased by the sample of their study. Schor et al. (2020) primarily interviewed hosts from the Boston area; none of the hosts they interviewed relied on Airbnb as their main source of income. In tourism regions, in contrast, Airbnb often constitute peoples’ main source of income (Farmaki et al., 2020). In addition, Schor et al. (2020) collected their data between 2013 and 2015, a time in which competition on the platform was less fierce, the platform’s policies more oriented toward pleasing hosts than guests, and guests’ expectations were generally lower (Farmaki and Kaniadakis, 2020). Thus, it is crucial to shed light on the hosts’ working conditions and consider how they impact these individuals’ life satisfaction.

One benefit of platform work that is frequently mentioned in the literature is the flexibility it provides relative to traditional employment (Vallas and Schor, 2020). Lampinen and Cheshire (2016) found that Airbnb hosts value the flexibility that Airbnb provides them in terms of when and how much they like to work. This flexibility can contribute to hosts’ life satisfaction, when it enables them to quit less flexible employment relations and to pursue valued identity projects. However, the extent of hosts’ flexibility can be compromised when service providers depend on the platform income for their livelihood (Schor et al., 2020). Moreover, the autonomy and flexibility of hosts is additionally constrained by the algorithmic management practices that Airbnb uses to manage hosts (von Richthofen and von Wangenheim, 2021). For example, Airbnb’s algorithm encourages hosts to answer booking inquiries as quickly as possible (Ravenelle, 2016). One of my interview participants explicitly complained about the pressure she feels to always have her phone by her side 24 h and 7 days a week, in case guests send her inquiries.

In addition, it is worth noting that any form of platform work carries a systematic risk for platform workers. Regardless of the exact terminology, micro-entrepreneurs or independent workers carry the entrepreneurial risk of their business. This is something Airbnb hosts had to face after the outbreak of the Corona pandemic, when guests canceled their bookings and global travel came to a stillstand. Meanwhile, some hosts had to cover expenses such as mortgages and salaries for employees (Farmaki et al., 2020).

One problematic aspect of hosting is that it blurs the boundaries between leisure and work. For instance, Zhang et al. (2019) found that, for some, the experience of hosting had a negative effect on their privacy and their sense of ease at home. Similarly, in their ethnographic work, Wilkinson and Wilkinson (2018) reported restricting themselves from engaging in routines such cooking and exercising when hosting guests because they felt uncomfortable or ashamed in front of them or because they wanted to avoid disturbing them. In one fieldnote, one of the authors documents the sentiment that she sometimes feels as if she were the guest. Throughout my netnographic fieldwork, I made similar observations. The following quote illustrates the extent to which the presence of guests can have a constraining influence on hosts:

When guests stay I literally tip toe around upstairs, keep the TV low, am always on at my 10 and 13-year old boys to talk quietly (poor them!), never have guests round in case they talk too loudly (!) or play music or have parties, (obviously!). (flaxhigh, reply to “Unsolicited advice from guest,” November 2, 2015).

The blurring of boundaries does not only concern the usage but also the decoration of spaces. Roelofsen (2018) insightfully observed that some hosts make adjustments to their home in order to cater to a particular type of guest.

### Hosting From the Perspective of Eudaimonic Wellbeing

Both my netnographic work and interviews suggest that being a provider on Airbnb can contribute to (and in some cases hinder) eudaimonic wellbeing. Below, I discuss the four dimensions of eudaimonia, namely, (1) self-realization, (2) personal growth, (3) a sense of purpose and meaning, and (4) relationships.

#### Self-Realization

For many service providers on Airbnb, hosting is not only a means to end, but a way to pursue self-realization and live in a manner consistent with their true self (Ryan and Deci, 2001; Ryff and Singer, 2008; Waterman et al., 2010). More specifically, my data analysis indicates that for such hosts, Airbnb is a means to pursue identity projects such as the identity of the bed and breakfast owner, the identity of the (micro-)entrepreneur, and/or the identity of the cosmopolitan. I will consider each identity in turn.

First, I found that some hosts value that Airbnb gave them the possibility of becoming hosts or bed and breakfast owners. The identity position of the bed and breakfast owner is prized. Like aspiring models (Parmentier and Fischer, 2015), the majority of bed and breakfast owners consider their work more of a lifestyle than job (Sweeney and Lynch, 2009). But while running a bed and breakfast requires substantial upfront investments, including the purchase or rental of an appropriate property, Airbnb enables hosts to rent out their extra space in their homes, to the delight of hosts who aspire this identity. Consider Sarah, for example, an American Airbnb host, who lives with her family in Maryland within commuting distance to Washington D.C. Hospitality has been always part of Sarah’s family’s lifestyle and she and her husband had long maintained the idea to open their own bed and breakfast: “In 2008 I wanted to go into the bnb business, but at the time trying to figure out all the licensing, etc., was too much. … So I’m very thankful that Airbnb made it so easy to get into … *the business I feel like I was meant to be in*” (Italics added). The quote shows that Sarah wanted to pursue the identity project of being an Airbnb host for a long time, but was thwarted off by the administrative hurdles involved. Thus, signing up on Airbnb, enabled her to realize her potential. Analyzing Sarah’s forum posts reveal several indicators in this regard. Her forum posts are often bursting with vitality, a positive affect associated with eudaimonic wellbeing (Ryan and Deci, 2001). Moreover, she *invested significant effort in the pursuit of excellence* (Waterman et al., 2010). Immediately after listing a room on Airbnb, she became an active participant on the Airhostsforum. In less than one and a half year, she made more than 2,500 forum posts, eagerly commenting and asking questions. In addition to taking the time to immerse herself in an online community around hosting, Sarah also invested considerable financial resources into her listing, expanding the room in her basement into a separate apartment. While Sarah may be an extreme case, she is no exception. Other studies, too, have found that there is a segment of providers on Airbnb, who identify strongly with their roles and derive gratification from being good hosts (Ikkala and Lampinen, 2015), join online communities around hosting (Farmaki and Kaniadakis, 2020), and experience self-fulfillment (Zhang et al., 2019).

There is another segment of providers on the Airbnb platform who identify less with the role of the host and instead see Airbnb as an opportunity to pursue the identity project of the (micro-)entrepreneur. Several studies found that some hosts value the entrepreneurial opportunity that Airbnb represents, professionalize over time, and eventually manage multiple properties (Ravenelle, 2016; Zhang et al., 2019; Buhalis et al., 2020; Farmaki and Kaniadakis, 2020). Similarly, I also found that many forum members professionalized over time and that a few ended up managing multiple properties. The following quote is illustrative of a provider on Airbnb, who eventually became a hospitality entrepreneur: “I’ve gone from sharing my home and flipping pancakes for (guests) (and sleeping on an air mattress in the basement for 2 years), to now owning three properties and growing a legit business” (superhostnyc, October 2016, reply to “Use Instant Book or host fees will increase from 3 to 5%!?”).

For some hosts, the identity value of hosting is that it helps them to pursue a cosmopolitan identity project (Ladegaard, 2018). Being cosmopolitan involves “that one is appreciative of the widest range and most culturally distant goods, places and tastes” (Üstüner and Holt, 2010). For aspiring cosmopolitans, the appeal of Airbnb is that it enables them to interact with people from foreign countries. The next interview quote from Pierre, a host from Paris, illustrates this:

Interviewer: (W)hat do you like the most about having guests with you? (…).

   Pierre: Meeting people from all over the world. (…) I like myself to travel and I travel to meet people (…) and doing Airbnb, people from all over the world are coming to my house (…) I had people from China, (…) I had a guy from Saudi-Arabia. Wow. I think I will never go to Saudi-Arabia. (…) Sometimes, I ask guests: “Can you bring me some food or some stuff from your country?” You know, like Kimchi from Korea (…) (Interview).

Pierre’s narrative clearly shows that he is pursuing a cosmopolitan identity project. He strives to travel, especially to culturally distant places, and is appreciative of the local culture—as illustrated by his request to guests to bring him local products. As a part-time teacher in France, however, Pierre’s identity project is limited in terms of time and money. Hosting guests in his apartment in Paris enables Pierre to get in touch with people from various countries and to learn about their cultures, including countries that he may never visit.

Before I conclude this section, it is important to note that some aspects of the Airbnb experiences can undermine the pursuit of the identity projects mentioned above. For example, given the time hosts spend cleaning and preparing the space between guests, some hosts feel that their work is less that of a host and more that of a cleaner (Buhalis et al., 2020). Moreover, realizing the identity project of a host requires the co-performance of the guest (von Richthofen and Fischer, 2019). This is why customer misbehavior (section “Hosting as a Source of Negative Affect”) can trigger negative effects that can go beyond negative affect and destabilize aspired identity projects.

#### Personal Growth

Zhang et al. (2019, p. 153) found that some hosts they interviewed mentioned the experience of personal growth as a positive consequence of hosting and that “they learned a great deal about customer service, communication, patience, and hospitality,” especially since being an Airbnb host involves dealing with people from different cultural backgrounds. My data analysis confirms their insight. There are plenty of forum posts, for example, which document how hosts initially struggled with certain guests, but ultimately found ways to cope with them. This often required acts such as speaking up and setting boundaries. Consider the following forum post:

… I’ve only been hosting for just over a year, I’ve had over 75 guests so feel I’ve learned something along the way… In the beginning I was always offering to do things for guests and asking them to let me know if anything wasn’t pleasing to them. And so they did! But usually in the reviews. So I became less solicitous (still welcoming and polite) but endeavored to appear less open to “advice” from guests. The pickiness stopped and the reviews stayed good. … (Wilburforce, May 27, 2016, reply to “Major guest fatigue”).

Some hosts even refer to hosting as a journey or an adventure, which indicates that hosting involves personal challenges they have to overcome on their way toward personal growth. In the following quote, for example, Sarah explicitly mentions that the challenges she encounters when interacting with guests help her to grow as a person:

Yes, this is where I’m growing as a person, too–trying to go from “this isn’t right!” to “OK, what’s this, and can we work around it.” Best part of being an airbnb host. (Sarah, July, 20, 2016, reply to “Guest using breakfast bar as his office”).

The following quote provides additional evidence of the personal growth some providers experience due to hosting and further indicates that some hosts are able to leverage the soft skills they acquire as hosts beyond Airbnb:

Introvert hermit female here! (…) Hosting has helped me become more relaxed around strangers and I’ve grown as a person in that way. It has also led me to have the guts to start up another rental business where I have to deal with people a lot. (eyeborg, July 10, 2016, reply to “Can someone who is not very outgoing be a successful host?”).

Consistent with Zhang et al. (2019), several of my interview participants found ways to leverage what they learned as hosts and to expand their business. Katy, for example, started out by hosting people in her extra room as a student but now leases an entire apartment in Zurich that she uses exclusively to rent out *via* Airbnb, making in times of high demand more money with the apartment than with her actual job. Another participant, Annatina, used some of the money she made through Airbnb to buy an apartment in Marseille and now employs her Airbnb knowledge to rent it out in order to cover a part of the mortgage.

#### Purpose and Meaning

Airbnb’s official mission is to create a world in which people can belong anywhere (Airbnb, 2014). Studies indicate that at least a portion of hosts have internalized and support this mission (Fitzmaurice et al., 2020; Makkar and Yap, 2020). Makkar and Yap (2020) found that some hosts in their sample were motivated by a “desire to practice morality,” wanted to promote a spirit of sharing in their communities, and felt as if they are helping people. Similarly, my data analysis indicates that at least a portion of Airbnb hosts perceive their work as meaningful, because they feel that they help their guests and that they contribute positively to their lives. Sophie, for example, explicitly stated that she “enjoy(s) that sort of level of helping people, it feels like I’m helping people.” Accommodating people, often from abroad, in their homes, hosts are in a natural position to help their guests. Airbnb tends to promote stories of hosts who went above and beyond for their guests in times of crisis (von Richthofen and Fischer, 2019). These stories often seem somewhat exaggerated and may happen not as frequently as Airbnb’s marketing communication suggests. However, I find that such instances do happen occasionally and give hosts the opportunity to help and, in consequence, derive meaning from it. The following story from one of my participants illustrates this:

(…) She said that she was really ill (…) so I went out to buy medicine for her (…). And then I made (…) Japanese chicken curry and then first she was like no, no, I don’t want it and then after a while, she had eaten it all and then she got a lot better and then she was like: “Oh my god I am really thankful that you kind of, pushed or insisted that I eat otherwise I wouldn’t have had the energy (to get better)” (Jennifer, interview).

While Jennifer did not explicitly articulate it, the fact that she shared the story indicates that she derived value from the fact that she was able to help her guest. Such instances are a part of what makes hosting meaningful to hosts like her.

Despite the evidence which indicates that many Airbnb service providers derive purpose and meaning from hosting, there is also evidence which points to the contrary. For example, Pierre (the host from Paris) generally supports the idea of home sharing. However, he struggles with the fact that by listing a room on Airbnb, he supports a company that has a reputation of contributing to developments he perceives critically, such as the gentrification of neighborhoods as well as mass tourism. Similarly, another interview participant expressed that she views Airbnb’s impact on cities critically.

#### Relationships

Hosting can be a source of relationships. Dozens of studies have documented that hosting can lead to pleasurable social encounters and in some cases even friendships with guests (e.g., Ikkala and Lampinen, 2015; Lampinen and Cheshire, 2016; Zhang et al., 2019; Farmaki and Kaniadakis, 2020; Fitzmaurice et al., 2020; Schor et al., 2020). Under certain circumstances, hosting may even help people to cope with loneliness (Farmaki and Stergiou, 2019). Since the social benefits hosts derive from hosting guests are well understood, I will focus here on the formation of relationships with other hosts as well as with individuals and organizations in their local communities. I will consider each source separately.

First, being Airbnb hosts can be an opportunity to get to know other Airbnb hosts, for example, by joining communities of practice (Wenger, 1998) around hosting. In addition to local communities that have developed over the years (Gallagher, 2017), there are numerous online communities (Buhalis et al., 2020; Farmaki and Kaniadakis, 2020) in which hosts interact and provide each other support, such as the Airhostsforum. It is not uncommon that forum members visit the Airhostsforum several times a day, chat about topics unrelated to hosting, and develop social bonds in the process. Some members expressed that they value this unexpected benefit of becoming Airbnb hosts: “I made good friends here on this forum” (Yana, November 5, 2015, reply to “Diary of a Happy Host?”).

Another source of positive social relationships are the local businesses that Airbnb hosts draw on to run their hospitality businesses: from shopping for groceries to employing cleaners, gardeners, and accountants (Ruiz-Correa et al., 2019; Farmaki et al., 2020). The following forum post humorously illustrates that being an Airbnb hosts can lead to new and unexpected communal relations: “You know you’re an Airbnb host when the local animal shelter knows you by name and looks forward to all the stained towels and sheets one donates monthly” (Ritz3, November 5, 2019, reply to “You know you are an Airbnb host when”).

Note, however, that while being an Airbnb hosts may help providers to socialize and form social bonds, it can also destabilize existing social bonds. One of my interview participants, Nanina, lives in a house wherein apartment owners share access to several resources and therefore have regular meetings. When she expressed her desire to list a room in her apartment on Airbnb, she experienced considerable push back from other tenants: “It is okay, with respect to the atmosphere (in the house), it is okay, but they are quite hostile toward Airbnb (…) I go way too far from their point of view with this entire situation.” Nanina’s quote indicates that becoming a host has damaged her relationship with her neighbors. This finding is consistent with emerging research on the impact of Airbnb on cities and neighborhoods (Dolnicar, 2019).

## Discussion

### Theoretical Implications

This article responds to call for research on the relationship between sharing economy participation and wellbeing (Eckhardt et al., 2019). More specifically, it contributes to the burgeoning literature on the relationship between participating in the sharing economy as service provider and wellbeing (Zhang et al., 2019; Buhalis et al., 2020). Overall, my findings indicate that participating in the sharing economy can both contribute to or hinder hedonic and eudaimonic wellbeing. In view to hedonic wellbeing, I identified three sources of pleasure and positive affect, namely, (1) the sociability involved in the host-guest interaction, (2) the practice of providing hospitality, and (3) positive feedback by guests. However, my findings showed that certain conditions can also hinder hosts’ wellbeing. Four conditions emerged as being especially relevant from my analysis: (1) customer misbehavior, (2) high volumes of guests, (3) negative feedback, and (4) income dependency. Moreover, I shed light on the relationship between sharing economy participation and life satisfaction, focusing on the role of income and the working conditions of being an Airbnb hosts. In the second part of the findings, I explored why and how hosting can contribute to or hinder hosts’ wellbeing from an eudaimonic perspective, focusing on four dimensions of eudaimonic wellbeing, namely, (1) self-realization, (2) personal growth, (3) purpose and meaning, and (4) relationships. The findings of this article make four contributions to the literature. First, this article provides a coherent account of the relationship between sharing economy participation and individual wellbeing. Second, it develops a number of aspects in more depth. While Schor et al. (2020) have insightfully observed that the experience of service providers depends to some extent on their dependency on the income generated through the platform, I showed in more detail how depending on the Airbnb income puts hosts under pressure to achieve high scores and provide a good experience to guests. Third, I identify a number of aspects related to hedonic wellbeing that the literature has not sufficiently highlighted, such as the volume of guests and negative feedback. Last but not least, this is the first paper that focuses explicitly on eudaimonic wellbeing and its various components in the sharing economy.

It is important to emphasize that no two hosts will experience their participation on the Airbnb platform alike. Experiences and the value that people derive from them are relativistic in the sense that they are personal and situational (Holbrook, 1999). The extent to which becoming an Airbnb host is a chance for self-realization and personal growth, for example, will depend substantially on the personality, identity projects, life goals, and personal circumstances of the host. To attract hosts and advertise its platform, Airbnb effectively promotes stories of hosts who seem to derive considerable meaning from hosting. In the “meet the host” series, for example, Airbnb showcases hosts, who seem by and large excited about the possibility to interact with guests, to identify with the role of the host, and to attribute considerable meaning to it (Airbnb, 2015). However, there are also many hosts who see Airbnb merely as a chance to generate some extra income (Ravenelle, 2016).

Given the dynamism of the development of the Airbnb platform and the sharing economy over the last decade, it is arguably futile to make any conclusions in terms of whether participating on the platform will ultimately contribute to the wellbeing of hosts. Instead, it is important to acknowledge that the wellbeing of Airbnb hosts—as well as the wellbeing of service providers in the sharing economy more generally—depends substantially on a number of contextual factors, such as the platform’s culture (Sundararajan, 2014; von Richthofen and Fischer, 2019) and governance (Farmaki and Kaniadakis, 2020; Frenken et al., 2020).

### Managerial Implications

The findings of this study have implications for both service providers and platforms in the sharing economy. I will consider each set of implications separately.

#### Implications for Service Providers

This study has several implications for service providers in the sharing economy in general and Airbnb hosts in particular. On a more basic level, it shows that participating in the sharing economy can both contribute to or hinder the wellbeing of hosts and service providers. Given that Airbnb offers probably some of the most favorable working conditions in the sharing economy (Schor et al., 2020), this study should serve as warning for consumers, who feel seduced by the emotional branding strategies of sharing economy platforms that promise them all the benefits of work such as money, sociability, and purpose, without any of the associated costs such as negative feedback and customer misbehavior.

In addition, I identify several conditions that can lead hosts to experience negative affect: (1) customer misbehavior, (2) high volumes of guests, (3) negative reviews, and (4) income dependency. I will discuss implications for each condition separately.

The types of customer misbehaviors which hosts in my study reported mirror those by guests in hotels (Harris and Reynolds, 2004). Studies indicate a number of strategies that hosts use to mitigate the risks of customer misbehaviors. For example, some hosts try to use their listing description and pricing to attract a particular type of guests and carefully select guests (Ikkala and Lampinen, 2015). During the stay, hosts can use various strategies to demarcate boundaries between shared and private space (Wilkinson and Wilkinson, 2018). Nevertheless, hosts will always face uncertainty when they allow strangers into their properties (Ladegaard, 2021).

The second condition, high volumes, is under the control of hosts as long as they do not fully depend on the Airbnb income. Based on this insight, it is recommended that hosts find a level of engagement on the platform that works for them. Moreover, hosts could consider to outsourcing some of the cleaning work or even the interaction with guests. There are already a number of agencies that offer assuming responsibility for everything including the booking process, welcoming of guests, and cleaning of the apartment.

An important insight of this study concerns the ambivalent role of reviews, which can be both a source of pleasure and positive affect as well as pain and negative affect. Hosts have substantial control over the guest experience (Benoit et al., 2017; von Richthofen and von Wangenheim, 2021) and they can influence guests’ satisfaction by managing expectations appropriately. However, guests may fail to read the carefully crafted listing descriptions by hosts, feel unsatisfied, and express their dissatisfaction publicly in reviews. Thus, it seems paramount that hosts find strategies to deal with reviews in a way that prevents harm to their wellbeing. This may involve employing various sorts of coping strategies. For example, hosts could engage in action-based coping by requesting that Airbnb removes unfair reviews, by publicly responding to reviews, and by seeking emotional support in online communities. Alternatively (or additionally), hosts may engage in more inner-oriented coping strategies, such as rationalizing the relative importance of, for example, one negative review in light of dozens of positive reviews. In either case, I observed that experienced hosts aspire to attribute less meaning to reviews and to avoid becoming too attached to them.

Fourth, providers should avoid relying entirely on the income generated through a particular platform. Doing so exposes them to the will of the platform and changes in policies. For example, service providers on Airbnb could consider listing their accommodations on several alternative peer-to-peer accommodation platforms.

#### Implications for Platforms

Sharing economy platforms depend on service providers as their co-producers (Dellaert, 2019)—without them, consumers would have no reason to use their platforms. Competition between brands to retain service providers on their platforms has been intensifying over the last years. Platforms such as Lyft and Uber compete by providing benefits such as online courses to drivers (“Drivers wanted: Ride-hailing apps try to burnish their image,” The Economist, 2019). It is therefore in the best interest of brands to ensure that participating on their platforms contributes positively to the wellbeing of service providers—especially since wellbeing is positively linked to outcomes such as organizational commitment (Jain et al., 2009). The managers of sharing economy platforms may especially benefit from considering the conditions that can hinder hosts’ wellbeing, namely, customer misbehavior, high volumes of guests, negative feedback, and income dependency.

First, it seems especially urgent that platforms find ways to prevent customer misbehavior. Platforms already use various means, such as the review system, to that end. However, the review system may be insufficient to discipline guests (customers), because negative reviews are more consequential for hosts (service providers), who depend on positive reviews to secure future bookings (Farmaki and Kaniadakis, 2020). Next to using reviews, platforms such as Airbnb also communicate norms and practices to customers (von Richthofen and Fischer, 2019; Bucher et al., 2020). For example, Airbnb nudges guests to write a couple of sentence about themselves and the purpose of their trip, when they contact hosts about a listing, in order to ensure a good fit between the guest and the host. But it seems that these tactics are insufficient to educate the masses of guests that have little or no knowledge of Airbnb’s history and the characteristics of the host-guest relationship. In addition, platform sometimes face tensions between conflicting goals. For example, Airbnb’s desire to prevent hosts from discriminating against guests based on ethnicity or country of origin, conflicts with one of the instruments the platform uses to familiarize hosts and guests with each other, namely obligatory profile pictures. Navigating such tensions will remain challenging and require a reflective approach from platforms.

Second, platforms such as Airbnb could consider sending service providers nudges when they have had a lot of volume and/or have not taken breaks for some time. However, platforms must carefully frame these reminders to avoid being perceived as patronizing. Third, my study indicated that negative feedback can be a source of negative affect. Evidently, the review system is a necessary instrument to create trust and enable exchanges between strangers (Parker et al., 2016). Nonetheless, platforms could find ways to reduce hosts’ dependency on reviews and the emotional stress associated with them (Zhang et al., 2019). For example, platforms could reduce the visibility and impact of individual reviews, so that one bad review cannot endanger a provider’s overall reputation. More generally, platforms could consider institutionalizing processes that enable both providers and consumers to challenge reviews they perceive as inaccurate or malicious.

Last but not least, platforms should consider being more transparent about both the benefits and costs as well as the chances and risks of platform work. A transparent approach would involve informing providers about the risks of depending on the platform income, which is still less reliable than the income from more stable forms of employment.

### Limitations and Opportunities for Future Research

This article has several limitations. One limitation of this article is that I did not directly assess the wellbeing of Airbnb hosts, but relied on the analysis of recurring themes indicative of hedonic and eudaimonic wellbeing in interviews and forum posts. Future research could use surveys to assess the wellbeing of Airbnb hosts more directly. Another promising avenue for future research is to explore the extent to which the wellbeing of hosts changes over time. Based on my own and prior studies (e.g., Buhalis et al., 2020), I would expect that hedonic wellbeing varies considerably over time. A related opportunity for future research is to explain the reasons for this variation, such as maturity on the platform or the characteristics of the platform’s governance. A second limitation of this study is that it considered only one sharing economy platform. Airbnb is practically synonymous with the sharing economy (Schor, 2016), but the experiences of Airbnb hosts are arguably better relative to those of workers on other sharing economy platforms (Schor et al., 2020). Thus, there is a need for systematic literature reviews and meta-analyses to compare the experiences of providers on different sharing economy platforms with respect to wellbeing. Last but not least, a final avenue for future research is to assess the relationship between different dimensions of wellbeing in the sharing economy. For example, how are facets of eudaimonic wellbeing, such as self-realization and personal growth, related to the experience of positive affect, negative affect, and life satisfaction of service providers in the sharing economy? Addressing these and other questions outlined above should give us a more nuanced understanding of the relationship between participating in the sharing economy as service provider and wellbeing.

## Data Availability Statement

The original contributions presented in the study are included in the article/Supplementary Material, further inquiries can be directed to the corresponding author/s.

## Author Contributions

The author confirms being the sole contributor of this work and has approved it for publication.

## Conflict of Interest

The author declares that the research was conducted in the absence of any commercial or financial relationships that could be construed as a potential conflict of interest.

## Publisher’s Note

All claims expressed in this article are solely those of the authors and do not necessarily represent those of their affiliated organizations, or those of the publisher, the editors and the reviewers. Any product that may be evaluated in this article, or claim that may be made by its manufacturer, is not guaranteed or endorsed by the publisher.

## Footnotes

1
https://airhostsforum.com
