# Supplementary material for: Happy Hosts? Hedonic and Eudaimonic Wellbeing in the Sharing Economy
Source: Front Psychol. 2022 Mar 16;13:802101. doi: 10.3389/fpsyg.2022.802101 (PMC8965736; doi:10.3389/fpsyg.2022.802101)
Supplement: Supplementary file 1 [file Table_1.DOCX]

Supplementary Material

| **Table 1: Profile of interview participants** | | | | | | |
| --- | --- | --- | --- | --- | --- | --- |
| No. | Pseudonym | Gender | Age | Occupation | Number of reviews | Listing type^1^ |
| 1 | Wolfgang | male | 63 | System administrator | 18 | PR |
| 2 | Christian | male | 42 | Professor | n.a. | EA |
| 3 | Annatina | female | 33 | Journalist | 48 | PR |
| 4 | Katy | female | n.a. | User experience designer | 110 | EA & PR |
| 5 | Nanina | female | n.a. | Care sector | 101 | PR |
| 6 | Franziska | female | 33 | Freelancer | 70 | EA |
| 7 | Sarah | female | 53 | Mother and home school teacher | 44 | EA |
| 8 | Sophie | female | 37 | Government worker | 34 | EA & PR |
| 9 | Jennifer | female | 30 | Lecturer | 34 | EA & PR |
| 10 | Manuela | female | 57 | Small business owner | 34 | EA |
| 11 | Pierre | male | 41 | Teacher | 72 | EA & PR |
| ^1^PR: Private room; EA: Entire accommodation | | | | | | |

| **Table 2: Interview protocol** | |
| --- | --- |
| Interview question | Probing question |
| Can you tell me a bit about yourself? | What do you do professionally?  How do you typically spend your free time? |
| How come you starting hosting on Airbnb? | When did you start hosting?  How many years have you been a host? |
| What do you like about hosting? |  |
| What do you dislike about hosting? |  |
| How do you interact with your guests? | How do you interact with your guests before/during the stay? |
| What is a good and successful guest stay for you? |  |
| What do you do with your Airbnb income? |  |
| What impact has hosting had on you? | What impact has hosting had on your life?  What impact has hosting had on the life of your family? |
